# Rice plants treated with biochar derived from Spirulina (*Arthrospira platensis*) optimize resource allocation towards seed production

**DOI:** 10.3389/fpls.2024.1422935

**Published:** 2024-09-18

**Authors:** Luana Vanessa Peretti Minello, Suelen Goettems Kuntzler, Thainá Inês Lamb, Cleo de Oliveira Neves, Emílio Berghahn, Roberta Pena da Paschoa, Vanildo Silveira, Jeferson Camargo de Lima, Cesar Aguzzoli, Raul Antonio Sperotto

**Affiliations:** ^1^ Botany Department, Graduate Program in Plant Physiology, Biology Institute, Federal University of Pelotas, Pelotas, Brazil; ^2^ Graduate Program in Biotechnology, University of Vale do Taquari - Univates, Lajeado, Brazil; ^3^ Graduate Program in Biotechnology, Federal University of Pelotas, Pelotas, Brazil; ^4^ Laboratory of Biotechnology, Bioscience and Biotechnology Center, State University of Northern Rio de Janeiro Darcy Ribeiro (UENF), Campos dos Goytacazes, Brazil; ^5^ Area of Knowledge in Exact Sciences and Engineering, Graduate Program in Materials Engineering and Science, University of Caxias do Sul (UCS), Caxias do Sul, Brazil

**Keywords:** agriculture, biostimulant, biofertilizer, cyanobacteria, microalgae, *Oryza sativa*, resource allocation, Spirulina

## Abstract

The use of biofertilizers is becoming an economical and environmentally friendly alternative to promote sustainable agriculture. Biochar from microalgae/cyanobacteria can be applied to enhance the productivity of food crops through soil improvement, slow nutrient absorption and release, increased water uptake, and long-term mitigation of greenhouse gas sequestration. Therefore, the aim of this study was to evaluate the stimulatory effects of biochar produced from Spirulina (*Arthrospira platensis*) biomass on the development and seed production of rice plants. Biochar was produced by slow pyrolysis at 300°C, and characterization was performed through microscopy, chemical, and structural composition analyses. Molecular and physiological analyses were performed in rice plants submitted to different biochar concentrations (0.02, 0.1, and 0.5 mg mL^-1^) to assess growth and productivity parameters. Morphological and physicochemical characterization revealed a heterogeneous morphology and the presence of several minerals (Na, K, P, Mg, Ca, S, Fe, and Si) in the biochar composition. Chemical modification of compounds post-pyrolysis and a highly porous structure with micropores were observed. Rice plants submitted to 0.5 mg mL^-1^ of biochar presented a decrease in root length, followed by an increase in root dry weight. The same concentration influenced seed production, with an increase of 44% in the number of seeds per plant, 17% in the percentage of full seeds per plant, 12% in the weight of 1,000 full seeds, 53% in the seed weight per plant, and 12% in grain area. Differential proteomic analyses in shoots and roots of rice plants submitted to 0.5 mg mL^-1^ of biochar for 20 days revealed a fine-tuning of resource allocation towards seed production. These results suggest that biochar derived from *Arthrospira platensis* biomass can stimulate rice seed production.

## Highlights

- Spirulina-derived biochar presents composition and structure suitable for use in agricultural systems.
- Exposure of rice plants to biochar for 20 days leads to proteomic changes in shoots and roots.
- Rice plants treated with biochar optimize resource allocation towards seed production.

## 1 Introduction

Rice (*Oryza sativa* L.) is one of the most important crops for meeting dietary needs and ensuring global food security. This crop covers 150 million hectares of planted area worldwide (Chen et al., 2020). Additionally, according to the World Agricultural Production report, global rice production in 2023/24 is projected to reach 520.9 million tons, surpassing previous crop yields (U.S. Department of Agriculture, 2024). In this production context, the American continent ranks second in global rice productivity, with Brazil being the largest producer, yielding 11.66 million tons in 2021 (FAO, 2023).

The high global demand for rice production necessitates the use of fertilizers, typically of chemical origin, to facilitate plant growth and development. However, the excessive and continuous application of chemical fertilizers can lead to environmental contamination, including soil mineral depletion and disruption of soil microbiota, as well as surface and groundwater pollution. Moreover, it contributes to greenhouse gas emissions, leaching, and poses risks to human health (Divya et al., 2023). Additionally, less than 50% of chemical fertilizers are effectively absorbed by plants, necessitating larger quantities for desired outcomes (Adhikari and Ramana, 2019). Therefore, the development of new products that minimize environmental impact, provide necessary nutrients for plant growth, and increase agricultural productivity becomes indispensable (Kawalekar, 2013).

Bioinputs such as biofertilizers and biostimulants are natural substances, providing an eco-friendly and economically viable alternative that aids in soil conditioning and enhances plant growth, ultimately increasing crop productivity. Various microorganisms have been utilized as bioinputs to stimulate plant growth and development (Pirttilä et al., 2021). Notably, microalgae/cyanobacteria like *Arthrospira platensis* exhibit key traits essential for sustainable agriculture, characterized by their photosynthetic nature, rapid growth rates, capability to thrive in wastewater, and utilization of atmospheric CO_2_ as a carbon source. Microalgae contain various compounds such as proteins, polysaccharides, lipids, minerals, and pigments that can enhance plant metabolic processes (Cao et al., 2023). The study by Lamb et al. (2023) demonstrated that indole compounds, siderophores, and exopolysaccharides present in microalgae act as biostimulants/biofertilizers in the development and seed production of rice plants.

The dry biomass of *A. platensis* contains approximately 45% C, 10% N, 12% P, 14% K, and other trace elements such as Mg, Ca, and Na, which are compounds of interest for plant growth (Binda et al., 2020). Therefore, *A. platensis* biomass has the potential to be directly applied in sustainable agriculture or used as a raw material to produce biofertilizers like biochar, the co-product resulting from the pyrolysis of any biomass (Huang and Gu, 2019). This compound is rich in carbon and, when applied to the soil, can increase water retention and ion exchange capacity, promote microbiota activity, minimize nutrient leaching, and regulate pH, thereby contributing to improved plant productivity (El-Naggar et al., 2019). Moreover, biochar can reduce phytotoxicity and stimulate plant metabolism, enhancing resilience capacity against biotic and abiotic stresses (Joseph et al., 2021).

Biochar derived from microalgae/cyanobacteria has been extensively studied for water and wastewater treatment, particularly in the removal of heavy metals, antibiotics, and dyes (Morais et al., 2024). However, there are still few studies demonstrating the advantages of microalgae/cyanobacteria biochar in soil enrichment, plant development, and productivity, especially in rice cultivation. Therefore, the aim of this study was to evaluate the molecular and physiological effects of biochar produced from *A. platensis* as biofertilizers and biostimulants on the development and productivity of rice plants.

## 2 Materials and methods

### 2.1 Material

*Arthrospira platensis* biomass was commercially acquired from Galena Química e Farmacêutica LTDA (Campinas, Brazil). Rice seeds (*Oryza sativa* L. ssp. *indica* cv. Puitá INTA-CL) were provided by Instituto Rio-Grandense do Arroz (IRGA) (Rio Grande do Sul, Brazil). All reagents used were analytical grade.

### 2.2 Biochar production

The powdered biomass of the cyanobacteria/microalgae *A. platensis* was acquired from Galena Química e Farmacêutica LTDA (Campinas, Brazil). For the preparation of the biochar, we followed the methodology described by Agnol et al. (2021), with minor modifications. The pyrolysis was conducted in a muffle furnace (SolidSteel, São Paulo, Brazil) at a heating rate of 10°C/min. To prepare the biochar, approximately 20 g of the biomass was placed in 55 mL porcelain crucibles, which were then placed in the muffle furnace for calcination at 300°C for 2 h. After carbonization, the biochar was cooled within the heating system until it reached room temperature. Subsequently, the biochar was manually pulverized into a fine powder using a mortar and pestle.

### 2.3 Characterization of biochar

The biochar was mounted on a metallic support using carbon tape and coated with gold for morphology analysis using scanning electron microscopy (SEM) (Carl Zeiss EVO-LS10, Germany). The chemical composition of the biochar was evaluated using the TESCAN VEGA3 model (Czech Republic), operating at 20 kV with a magnification of 100x, appraised through energy dispersive spectroscopy (EDS), which was integrated with SEM using the Bruker Nano XFlash Detector 6-10. Additionally, the samples were analyzed by X-Ray Fluorescence spectrometer (XRF) (Shimadzu, EDX 7000) for elemental mapping. Changes in the molecular structure of the biochar were assessed using Fourier-Transform InfraRed (FTIR) spectroscopy. The spectrophotometer (Shimadzu IRAffinity-1, Japan) was utilized to detect functional groups, recording spectra between 400 and 4000 cm^-1^ with a spectral resolution of 4 cm^-1^. Surface area was determined using the Brunauer, Emmett, Teller (BET) method, and porosity was assessed using the Barret, Joyner, Halenda (BJH) method on a Gemini VII 2390 surface analyzer (Micromeritics, USA). Prior to analysis, the sample was dried at 180°C for 3 h in a nitrogen atmosphere, following the procedure outlined by Reis et al. (2021). pH analysis of the biochar was conducted using a digital pH meter at 25°C (Digimed, Brazil).

### 2.4 Plant cultivation

Seed asepsis was conducted by immersing the seeds in a solution comprising 60% distilled water, 40% sodium hypochlorite (2%), and three drops of neutral detergent for 5 min. Subsequently, the seeds were rinsed five times with distilled water for 5 min each. Following asepsis, rice seeds were germinated in Olen boxes (model K31-1000-5), containing a complete nutrient solution (pH 5.4) as described by Ricachenevsky et al. (2011). The nutrient solution was changed three times per week. The boxes were placed in a BOD-type growth chamber set at 25°C with a photoperiod of 16 h light (200 μmol m^−2^ s^−1^) and 8 h dark for 20 days to facilitate seed germination and seedling development. After this initial period, the seedlings were transferred to a growth room maintained at 25°C with a photoperiod of 16 h light (200 μmol m^−2^ s^−1^) and 8 h dark for an additional 20 days.

### 2.5 Biochar treatment and plant growth evaluation

The experiment consisted of four treatments: a control condition with only nutrient solution, as described by Ricachenevsky et al. (2011), and three conditions with different concentrations of biochar (0.02, 0.1, and 0.5 mg mL^-1^) added to the complete nutrient solution. The pH of the nutrient solutions was adjusted to 5.4 after the addition of biochar. The experiment was conducted in biologically independent triplicates. Plant collections were carried out after 10 and 20 days of treatment with biochar, approximately at the V4 vegetative stage, according to Counce et al. (2000). At each collection, 15 plants from each condition were harvested for growth parameter analysis. Following the second collection, rice plants from each treatment were transplanted into 8 L pots containing Tropstrato substrate (Vida Verde). The water level in the pots was established after 5 days using distilled water, and the plants remained in a greenhouse until the end of the cycle (approximately 125 days) for productivity analyses.

### 2.6 Productivity parameters

Plants were analyzed based on key components recommended for the culture, including the time to phase transition (vegetative vs. reproductive), which involved counting days to reach stages R3 (panicle emergence), R4 (anthesis), and R5 (grain filling), according to Counce et al. (2000). Other parameters assessed included the plant height, number of tillers, number of seeds per plant, percentage of full seeds per plant, weight of 1,000 full seeds, seed weight per plant, grain length, and grain area. Grain length was measured using a digital caliper (DIGIMESS-100.172, Brazil), and the grain area was calculated using ImageJ software. All data were presented as the means and standard errors of at least 15 independent biological replicates.

### 2.7 Protein extraction and digestion

Proteins were extracted from 250 mg of fresh shoots and roots from rice plants submitted to control condition or to 0.5 mg mL^-1^ biochar for 20 days by pulverizing them in the presence of liquid nitrogen until a fine powder was formed. Subsequently, proteins were extracted and purified using the solvents provided in the Plant Total Protein Extraction Kit (Sigma-Aldrich, USA), following the manufacturer’s recommendations. The protein concentration was determined using the 2-D Quant Kit (Cytiva, USA). Protein digestion was carried out using 100 μg of protein from each sample and trypsin as a denaturing agent. Initially, the samples were precipitated using a methanol/chloroform solution to remove possible detergent fragments, following the protocol outlined by Nanjo et al. (2012). After precipitation, the samples were resuspended in a 7 M urea/2 M thiourea buffer, and tryptic digestion of proteins (V5111; Promega, USA; final enzyme-to-protein ratio of 1:100) was performed using the filter-aided sample preparation (FASP) method (Wiśniewski et al., 2009), with modifications (Burrieza et al., 2019). The resulting peptides were quantified using the protein and peptide method at 205 nm using a NanoDrop 2000c spectrophotometer (Thermo Fisher Scientific, USA).

### 2.8 Mass spectrometry analysis

Mass spectrometry was performed using a nanoACQUITY ultra-performance liquid chromatograph (UPLC) coupled to a Q-TOF SYNAPT G2-Si instrument (Waters, UK) as described by da Paschoa et al. (2024).

### 2.9 Proteomic data analysis

Spectra processing and database search conditions were performed according to da Paschoa et al. (2024). For data analysis, the proteome of the species *Oryza sativa* (ID: UP000059680), available on UniProtKB (https://www.uniprot.org/), was used. Label-free quantification analysis was performed according to da Paschoa et al. (2024). For the comparative analysis, only the proteins present or absent (for unique proteins) in the three biological replicates were accepted for differential abundance analysis. Comparative analyses were performed on samples treated with biochar relative to control samples. Data were analyzed by Student’s *t*-test (two-tailed). Proteins with a p-value < 0.05 were deemed up-accumulated if the Log2 of the fold change (FC) > 0.6, and down-accumulated if the Log2 of the FC < -0.6. The mass spectrometry proteomics data have been deposited to the ProteomeXchange Consortium via the PRIDE (Perez-Riverol et al., 2022) partner repository with the dataset identifier PXD051225.

### 2.10 Protein-protein interaction network

A protein-protein interaction (PPI) network analysis was conducted to unravel rice responses to 20-days biochar exposure. PPI networks were constructed via the metasearch tool STRING 11.5 (https://string-db.org/) utilizing all differentially abundant proteins identified through MS/MS using the *Oryza sativa* database, adding 50 shell proteins (directly associated with our input proteins), and with the “Textmining” option disabled. Given that an additional shell of proteins was used as input together with the proteins identified by MS/MS, a minimum required interaction score of high confidence was chosen (0.700). To pinpoint high-degree proteins pivotal in the PPI network as predicted by STRING, we employed Cytoscape software 3.9.1 (https://cytoscape.org/), and subsequently utilized the Cytoscape plugin Cytohubba 0.1 (https://apps.cytoscape.org/apps/cytohubba).

### 2.11 Statistical analysis

For all data, except the proteomics, Shapiro–Wilk (for normality) and Levene (for homogeneity of variance) tests were performed on the residuals of the fitted model. All data demonstrated normal distribution and were statistically compared (control *vs.* biochar concentrations) utilizing One-Way ANOVA followed by Tukey’s test (p ≤ 0.05) in the case of homogeneous variance or followed by Dunnett’s C test (p ≤ 0.05) in the case of heterogeneous variance. SPSS Base 23.0 for Windows (SPSS Inc., USA) was used for statistical analysis.

## 3 Results and discussion

### 3.1 Morphological and physicochemical characterization of biochar

**Figure 1:**
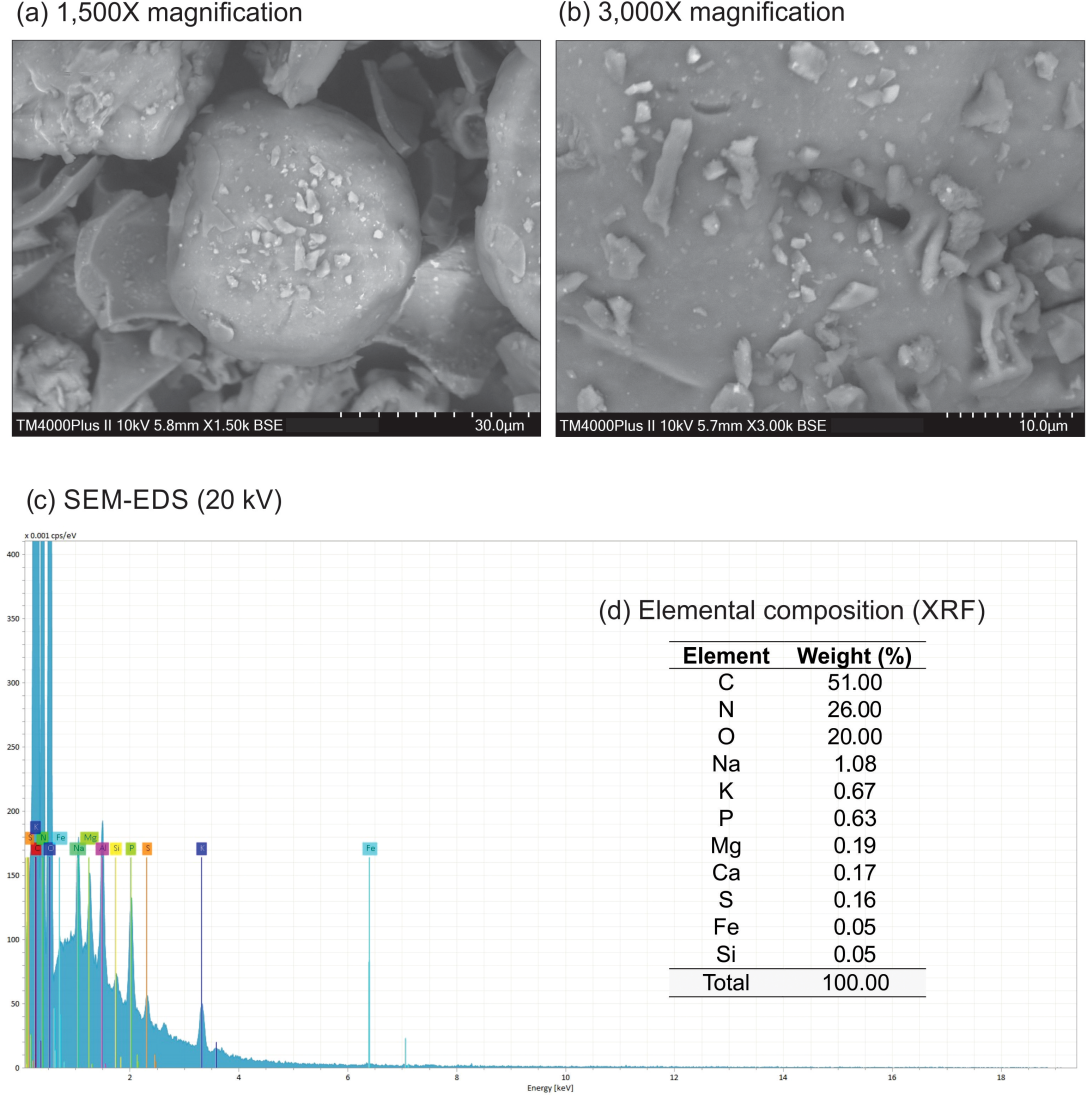
SEM images **(A, B)** at two different magnifications (1,500x and 3,000x), SEM-EDS 20 kV spectra **(C)**, and elemental composition **(D)** by XRF of biochar derived from *Arthrospira platensis*.

In the SEM images, the biochar exhibits a heterogeneous morphology characterized by irregular structures, a rough surface, and the presence of particles of various sizes and shapes (**Figures 1A, B**). These features likely indicate the presence of amorphous organic regions affected by the thermal decomposition of the raw material, as the carbon structure typically remains unaffected by pyrolysis at 350°C (Mendonça et al., 2017). Similar observations were made by Ma et al. (2022), who also noted a heterogeneous morphology and rough surface in Spirulina biochar. EDS analysis indicated high levels of C, N, and O (51.00, 26.00, and 20.00 wt%, respectively - **Figure 1C**). Additionally, several minerals were identified using XRF analysis (Na: 1.08; K: 0.67; P: 0.63; Mg: 0.19; Ca: 0.17; S: 0.16; Fe: 0.05; and Si: 0.05 wt% - **Figure 1D**). Such elemental composition may contribute to the formation of its rough surface and positively influence seed germination. Sun et al. (2023) reported an 11% increase in the germination rate of *Nasturtium officinale* seeds treated with microalgae biochar compared to the control group. Additionally, Binda et al. (2020) identified higher levels of Na, K, and Mg in *Arthrospira* sp. biochar compared to *Chlorella vulgaris* biochar.

According to Law et al. (2022), alkali and earth alkali metals presented in biochar composition may increase the pH of the biochar. Therefore, the mineral composition of the Spirulina-derived biochar may have contributed to the observed alkalinity (pH 7.8). pH is a critical parameter in considering the application of *A. platensis* biochar in agriculture, as it can be utilized for soil correction purposes. Furthermore, biochar with a high alkaline pH indicates a high ash content, which can lead to improved soil structure, microbial biodiversity, rhizobia nodulation, and increased concentrations of minerals crucial for plant development (Gryta et al., 2023). Generally, biochar has an alkaline pH due to synthesis conditions, biomass used, and inorganic elements comprising it, such as phosphates and ashes (Chen et al., 2019a). Despite its basic characteristic, studies suggest the addition of biochar even in alkaline soils as a mitigative measure to increase carbon sequestration, improving their physicochemical properties and, consequently, the development of cultivated plants (Abrishamkesh et al., 2015; Mete et al., 2015). Other studies show that the application of alkaline biochar does not increase the pH of alkaline soils; instead, it may induce a decreasing trend in pH, especially with higher rates of biochar application (Zhang and Liu, 2012). The use of biochar in sandy or acidic soils has been shown to significantly increase plant productivity (Dai et al., 2020). However, it’s important to conduct a prior evaluation of soil composition to determine the most suitable biochar, as negative effects on plants may arise if the properties of the biochar and soil are not considered together.

The *A. platensis* biochar produced through slow pyrolysis exhibited a yield of 55% of the original *A. platensis* weight. The high yield is attributed to the low temperature (300°C) employed in this study during the production process, as this parameter depends on both temperature and residence time. Sun et al. (2023) demonstrated that microalgae biochar yields decreased from approximately 37% to 32% with an increase in pyrolysis temperature from 450 to 700°C. The pyrolysis of microalgae biomass begins with dehydration at temperatures below 200°C, followed by the decomposition of carbohydrates, lipids, and proteins between 200 and 550°C (Sun et al., 2022). The high production yield of biochar is considered highly feasible and reliable for agricultural use when compared to other methods, as described by Yu et al. (2017).

**Figure 2:**
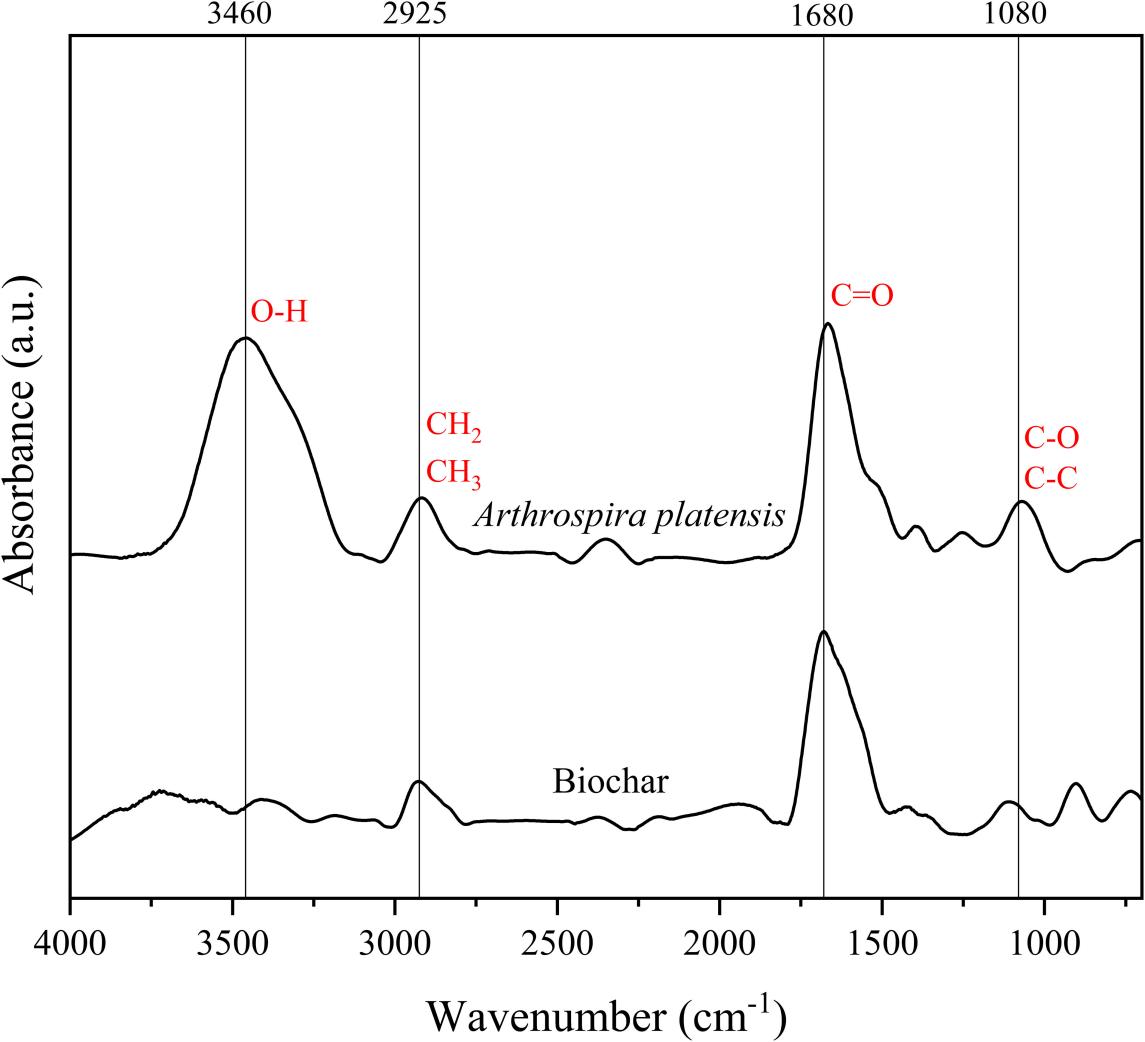
FTIR spectra of biomass and biochar from *Arthrospira platensis* showing the putative functional groups.

FTIR analysis was conducted to identify the different functional groups present in the *A. platensis* biomass and biochar (**Figure 2**). In the biomass spectrum, a broad band around the peak at 3460 cm^-1^ is observed, representing the hydroxyl (O-H) group (Cheng et al., 2023), attributed to compounds such as phenols and amine groups (Binda et al., 2020), and polysaccharides (Wang et al., 2024). The peak at 2925 cm^-1^ is associated with asymmetric C-H vibration (Wang et al., 2022), indicating the presence of hydrocarbons (CH_2_ and CH_3_ groups - Cheng et al., 2023), typical of polysaccharides structures, mainly cellulose fiber (Wang et al., 2024). At 1680 cm^-1^ peak we detected a broad band present in both spectra, typical of C=O bond stretching of the amide of polypeptides/proteins (Chen et al., 2019b). The peak around 1080 cm^-1^ indicates the coupling of the C-O or the C-C stretching modes (Tas et al., 2022), suggesting the presence of phosphate compounds (Choi et al., 2020) or polysaccharides (Wang et al., 2022; Cheng et al., 2023). The absence or low intensity of peaks representing functional groups in the biochar indicates that pyrolysis causes modification in the structure and chemical bonds of the compounds. In some cases, high pyrolysis temperatures can also lead to the formation of aromatic compounds, such as the peak at 880 cm^-1^ observed in the biochar spectrum (Pena et al., 2023). Our results are similar to those reported in the literature for other microalgae/cyanobacteria biomasses (Cheng et al., 2023; Ladjal-Ettoumi et al., 2024). Therefore, these compounds present in *A. platensis* biochar could potentially participate in the biostimulation of rice plants.

The *A. platensis* biochar exhibited a surface area of 0.5236 m² g^-1^, a pore volume of 0.000298 cm³ g^-1^, and an average pore size of 2.039 nm. These parameters, including porosity and specific surface area, play crucial roles in improving soil properties and promoting water adsorption. They are significantly influenced by the pyrolysis temperature, with higher temperatures resulting in increased surface area and pore volume (Gan et al., 2018). For instance, the surface area of Spirulina biochar increased from 2.2 to 4.0 m² g^-1^ when the production temperature was raised from 300 to 400°C. A similar trend was observed regarding pore volume (Piloni et al., 2021).

The pore distribution analysis revealed a heterogeneous surface of the biochar, consisting of micropores (0-2 nm) and mesopores (2-40 nm). This porous structure offers numerous benefits, including increased water retention capacity, gas and nutrient adsorption, and greater soil carbon stability and storage (Amin et al., 2016). Consequently, the characterization of biochar highlights the potential of the microalgae/cyanobacteria *A. platensis* to produce biomaterials with intriguing physicochemical and structural properties for various applications, such as soil fertilizers and plant growth stimulants. Moreover, the morphology, presence of mineral salts, and highly porous structure of biochar may have contributed to enhanced nutrient availability for plants, thereby increasing rice plant productivity, as demonstrated in the subsequent sections.

### 3.2 Influence of biochar on the growth and development of rice plants

**Figure 3:**
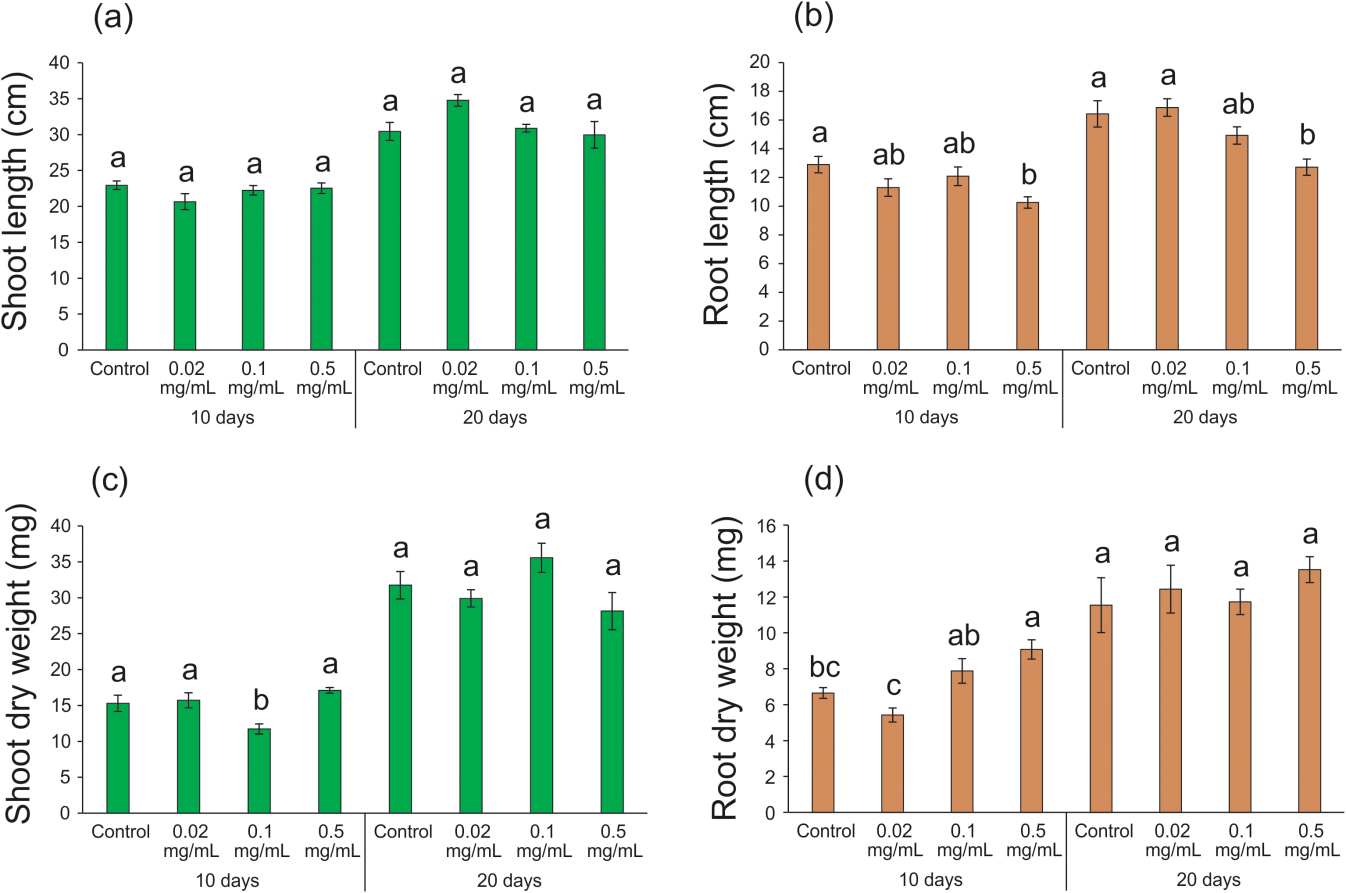
Growth parameters of rice plants treated with 0.02, 0.1, and 0.5 mg mL^-1^ of Spirulina-derived biochar for 10 and 20 days. Shoot length **(A)**, root length **(B)**, shoot dry weight **(C)**, and root dry weight **(D)**. Bars represent the mean ± SE (n = 8). Different letters indicate statistically significant differences (P ≤ 0.05) between tested conditions (Control, 0.02, 0.1, and 0.5 mg mL^-1^ of biochar) in each exposure time (10 and 20 days).

Improved absorption of nutrients and water by plants is attributed to the increased length of primary and adventitious roots (Cochavi et al., 2020). Additionally, mass increment is a crucial factor for plant development, as it contributes to increased leaf number and area, enhancing light interception efficiency and photosynthate metabolism in plants (Liu et al., 2021b). In our study, no significant differences were observed in shoot length after 10 (*F* = 1.555; df = 3, 27; p = 0.223) and 20 days (*F* = 2.332; df = 3, 25; p = 0.100) of exposure to different biochar concentrations when compared to the control condition (**Figure 3A**). However, a significant reduction (20-23%) in root length was detected after 10 (10.26 ± 0.40; *F* = 4.142; df = 3, 25; p = 0.016) and 20 days (12.71 ± 0.57; *F* = 6.959; df = 3, 26; p = 0.001) of exposure to 0.5 mg mL^-1^ biochar in relation to the control condition (10 days: 12.90 ± 0.57; 20 days: 16.43 ± 0.92 - **Figure 3B**). The absence of a significant effect on shoot length and the observed reduction in root length were not entirely unexpected, as previous studies have reported conflicting results regarding the impact of biochar treatment on these parameters (Liu et al., 2021a). While some studies have shown an increase in shoot and/or root length following biochar treatment (An et al., 2022; Han et al., 2023), others have reported a decrease (Lakitan et al., 2018; Yin et al., 2023; Ruan and Wang, 2024). Despite the various benefits associated with biochar incorporation into soil or hydroponics, outcomes can be highly variable due to factors such as the origin/quality of the raw material, the production process of the biochar, the application dosages of biochar, the genotype/cultivar/species tested, as well as the management practices and irrigation systems used in plant cultivation (Joseph et al., 2021). It is well-established that high concentrations of biochar applied to soil can lead to toxic effects (Zafar et al., 2023). For instance, concentrations as high as 40 mg mL^-1^ inhibited the growth of *Arabidopsis thaliana* roots by up to 55%, attributed to the toxic accumulation of molecules in the root meristematic nucleus. This accumulation can disrupt the transcription levels of genes involved in auxin biosynthesis, a critical hormone for root development, ultimately hindering plant growth (Yan et al., 2021). However, in our study, the highest concentration used (0.5 mg mL^-1^) was likely insufficient to induce toxicity. Therefore, the observed lack of stimulatory effects on shoot and root length may signify an optimization of resource allocation, a notion supported by the proteomic data presented in subsequent sections.

Similar to the shoot length data, the exposure of rice plants to different biochar concentrations for 20 days did not result in significant changes in shoot dry weight (*F* = 2.614; df = 3, 27; p = 0.072). After 10 days of exposure to 0.1 mg mL^-1^ of biochar, we detected a slight decrease in shoot dry weight (11.74 ± 0.70; *F* = 7.350; df = 3, 23; p = 0.001) when compared with the control condition (15.30 ± 1.12 - **Figure 3C**). Surprisingly, when rice plants were exposed to 0.5 mg mL^-1^ of biochar for 10 days, we detected the highest root dry weight (*F* = 8.337; df = 3, 13; p = 0.002 - **Figure 3D**), despite the same treatment resulting in the lowest root length (**Figure 3B**). This suggests that the increase in dry weight may be attributed to the thickening of the cell wall of root cells, probably leading to an increase in the diameter of these roots, rather than growth in length. Therefore, it is noteworthy that the primary growth and developmental effects of biochar application in rice plants are related to root tissues. While many studies linking biochar use to enhanced root development focus on increased root length and improved uptake of immobile nutrients in soils (Abiven et al., 2015; Huang et al., 2021), it is important to recognize that increasing root diameter can also contribute to enhancing root surface area. This strategy may be particularly relevant for plants grown in a hydroponic system like ours. To the best of our knowledge, this is the first report of enhanced thickening of the root system in plants subjected to a brief exposure to biochar. After 20 days, no significant differences were observed among the treatments for root dry weight (*F* = 0.445; df = 3, 20; p = 0.724 - **Figure 3D**), suggesting that the increase in root thickening is a rapid response to the biochar application.

**Figure 4:**
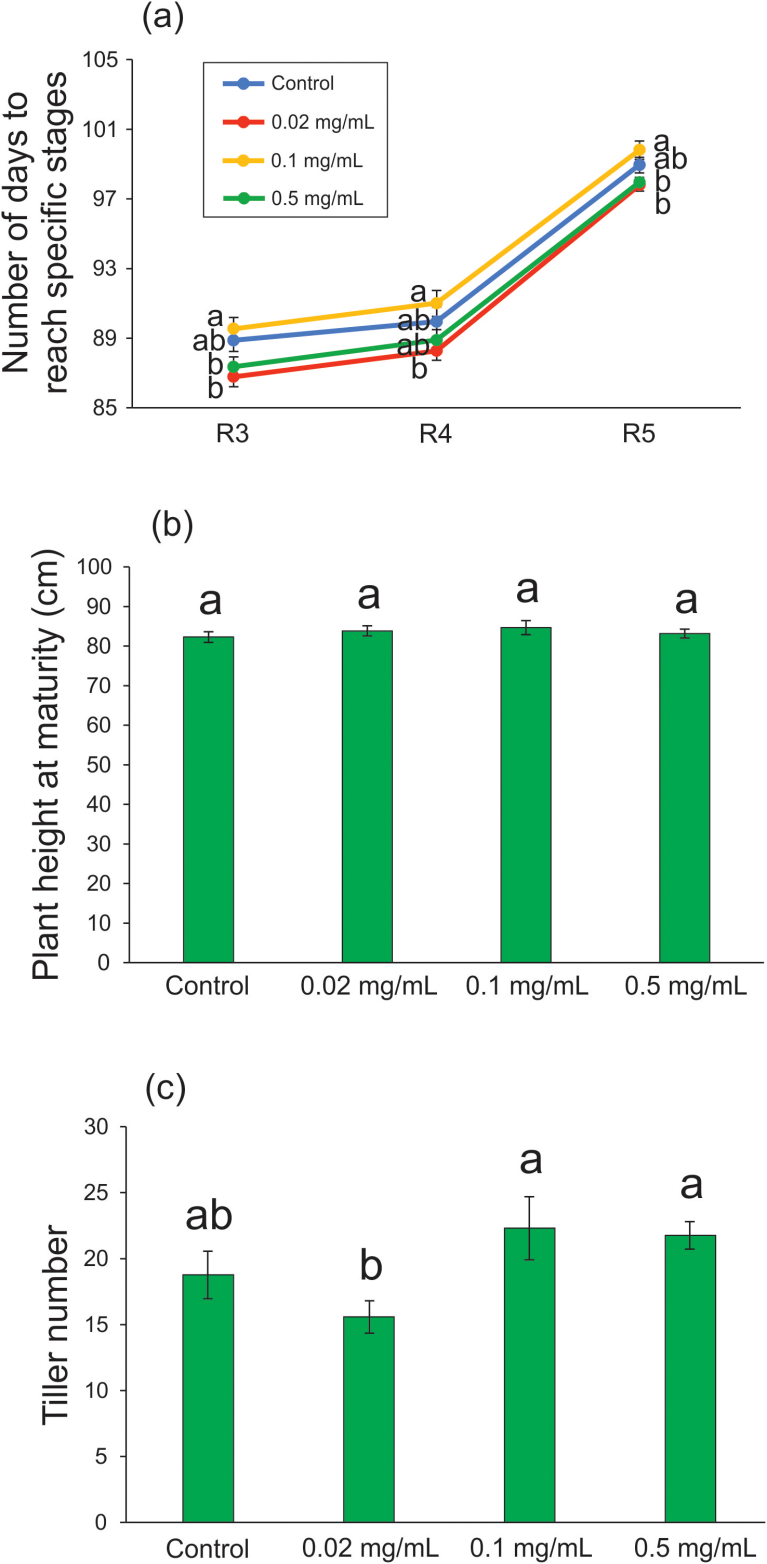
Developmental characteristics of rice plants treated with 0.02, 0.1, and 0.5 mg mL^-1^ of Spirulina-derived biochar for 20 days during the vegetative stage. Number of days to reach specific developmental stages **(A)**, plant height at maturity **(B)**, and tiller number **(C)**. Bars represent the mean ± SE (n = 15). Different letters indicate statistically significant differences (P ≤ 0.05) between tested conditions (Control, 0.02, 0.1, and 0.5 mg mL^-1^ of biochar). In **(A)**, different letters indicate statistically significant differences (P ≤ 0.05) between tested conditions (Control, 0.02, 0.1, and 0.5 mg mL^-1^ of biochar) in each developmental stage: R3 (panicle emergence), R4 (anthesis), and R5 (grain filling).

After the plants were transplanted to the substrate and maintained under greenhouse conditions, compared with the control condition, we were unable to detect any effect of biochar exposure on the number of days to reach specific developmental stages (R3: *F* = 4.510; df = 3, 61; p = 0.006; R4: *F* = 3.013; df = 3, 64; p = 0.036; R5: *F* = 5.209; df = 3, 64; p = 0.003 - **Figure 4A**), plant height at maturity (*F* = 0.366; df = 3, 64; p = 0.778 - **Figure 4B**), and tiller number (*F* = 3.910; df = 3, 62; p = 0.013 - **Figure 4C**), regardless of the tested concentration. According to Lakitan et al. (2018), the application of wood biochar at rates up to 1.2 Mg ha^-1^ in rice cultivation increased tillering and rice yield without affecting plant height at maturity. In the following section, we demonstrate that a 20-day exposure of rice plants to 0.5 mg mL^-1^ Spirulina-derived biochar during the vegetative stage can increase seed production without interfering with the tiller number.

### 3.3 Influence of biochar on rice seed production

**Figure 5:**
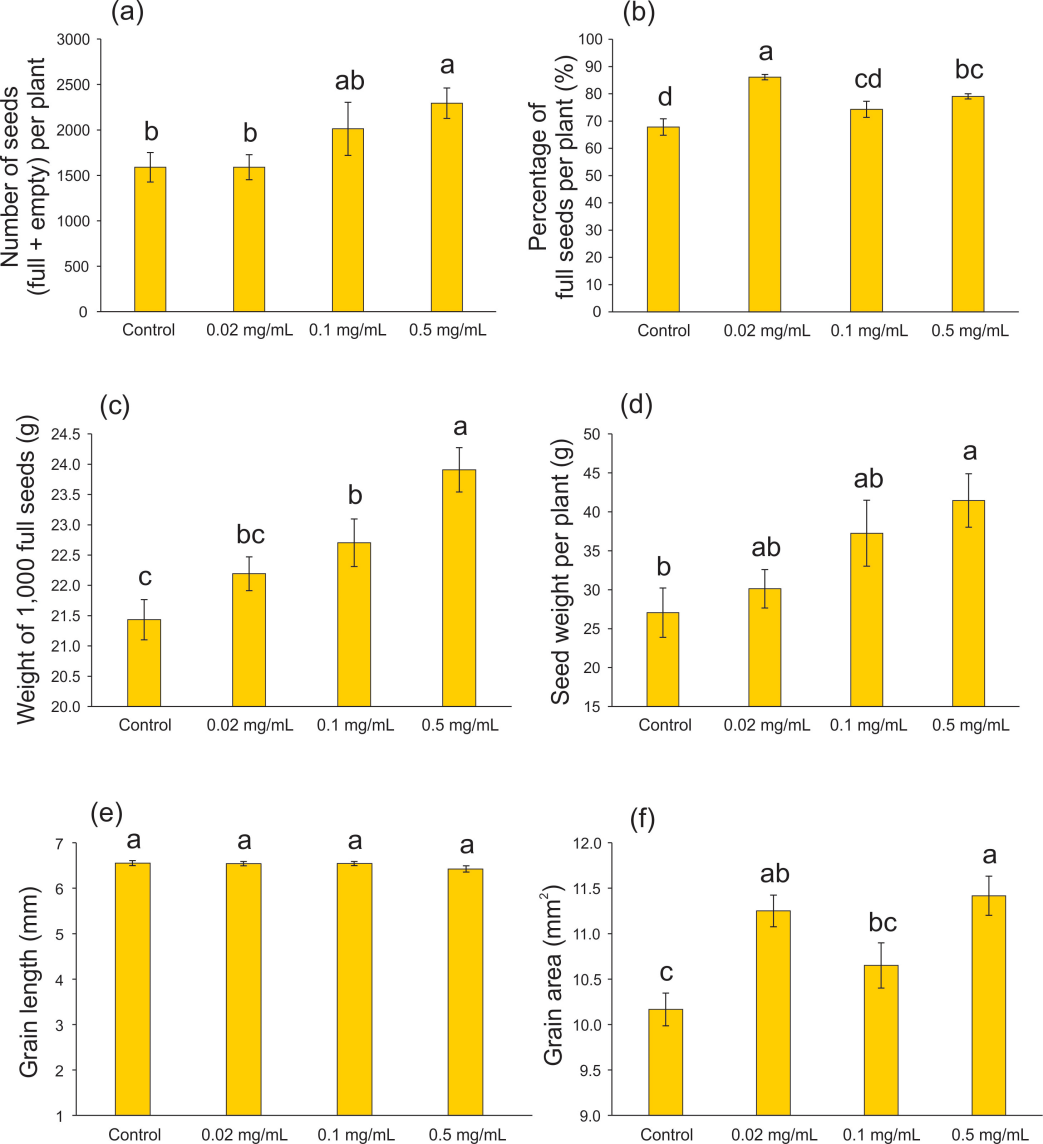
Seed characteristics of rice plants treated with 0.02, 0.1, and 0.5 mg mL^-1^ of Spirulina-derived biochar for 20 days during the vegetative stage. Number of seeds (empty + full) per plant **(A)**, percentage of full seeds per plant **(B)**, weight of 1,000 full seeds **(C)**, seed weight per plant **(D)**, grain length **(E)**, and grain area **(F)**. Bars represent the mean ± SE [n = 15 in **(A, B, D)**; n = 50 in **(C)**; n = 30 in **(E, F)**]. Different letters indicate statistically significant differences (P ≤ 0.05) between tested conditions (Control, 0.02, 0.1, and 0.5 mg mL^-1^ of biochar).

At the full maturity stage, seed production was evaluated to assess the effect of 20 days’ exposure of rice plants to Spirulina-derived biochar. Compared with the control condition, the highest biochar concentration (0.5 mg mL^-1^) resulted in an increased number of seeds (empty + full) per plant (*F* = 3.139; df = 3, 59; p = 0.032 - **Figure 5A**), percentage of full seeds per plant (*F* = 13.911; df = 3, 63; p < 0.001 - **Figure 5B**), weight of 1,000 full seeds (*F* = 9.090; df = 3, 196; p < 0.001 - **Figure 5C**), seed weight per plant (*F* = 4.115; df = 3, 61; p = 0.010 - **Figure 5D**), and grain area (*F* = 7.697; df = 3, 116; p < 0.001 - **Figure 5F**), while grain length was not affected by the biochar application (*F* = 1.262; df = 3, 116; p = 0.291 - **Figure 5E**). Similar results were found using biochar produced from different biomasses. Lakitan et al. (2018) showed that applying wood biochar to rice plantation at rates up to 1.2 Mg ha^-1^ increase several rice yield components, as number of grains per panicle, panicle density, percentage of filled grain, and weight of 1,000 grains. Rice straw biochar application increased the grain yield of rice plants after 6-year successive application (1.5 t ha^-1^ year^-1^ - An et al., 2022). Also applying rice straw biochar (20 g kg^-1^ soil) during the entire growing season, Chen et al. (2023) showed that rice plants increased the effective panicle, grain number per panicle and seed setting rate. However, it decreased the 1,000-grain weight, resulting in an increase in yield (Chen et al., 2023).

It is important to note that most of the research on biochar involves long and/or continuous applications (Huang et al., 2018; Xu et al., 2022), spanning up to six years (An et al., 2022). To the best of our knowledge, this is the first study to demonstrate a beneficial effect of short-term biochar application (20 days during the vegetative stage) on rice seed production. The Puitá INTA-CL cultivar (used in our work) is characterized by a medium cycle, short stature, and high tillering capacity. In field conditions, this genotype demonstrates its maximum productive potential, with a weight of 1,000 grains reaching 25.7 g. However, achieving this potential relies heavily on the soil and climatic conditions of the cultivated area, which are rarely optimal in arable areas, even with ideal soil profiles (He et al., 2023). In this study, we demonstrated that the incorporation of microalgae/cyanobacteria biochar into the growing medium enabled plants to approach very close to the maximum productive potential, reaching approximately 24 g. This can significantly enhance both the quality and quantity of grains in a smaller planting area. Moreover, the utilization of microalgae biochar can reduce fertilizer costs in crop management when combined with other cost-effective sources of N for soil application, thanks to the presence of micropores that facilitate the slow release of essential nutrients for plants (Gonçalves et al., 2023; Yadav et al., 2023).

Another promising application is the addition of biochar to crop rotation areas in conjunction with green manure, as it helps mitigate greenhouse gas emissions and provides C availability for subsequent crops (Zhang et al., 2023). The food crisis, wars, and climate change have posed challenges to global agricultural production. In addition, agricultural soils have been experiencing degradation and leaching over the past 100 years. It is estimated that food demand will increase by approximately 70% by 2050 (Kopittke et al., 2019). Therefore, the development of environmentally friendly, low-cost, slow-release, and highly efficient biofertilizers is necessary. In this context, the use of Spirulina-derived biochar may emerge as an efficient mitigating measure for soil recovery and nutrient delivery to plants, as well as reducing the effects of climate change through long-term C sequestration and contributing to global food security.

The productivity of rice grown in soils with biochar largely depends on the type of management. In rainfed rice cultivation, an increase of 10.6% in productivity was observed, while flooded rice showed an increase of 5.6% (Ye et al., 2020). This difference can be attributed to the ability of biochar to retain water and alkalinize the soil, resulting in less significant responses under ideal water conditions. Other studies have reported an increase in the yield of vegetables such as broccoli, tomato, lettuce, coriander, basil, spinach, and pepper grown with biochar (≤ 20 t ha^-1^) combined with inorganic fertilizers (Choi et al., 2018; Edussuriya et al., 2023). This effect is attributed to the ability of biochar to retain and improve biological N_2_ fixation and make K available to plants, contributing to up to a 15% increase in productivity (Rondon et al., 2007; Güereña et al., 2013; Van Zwieten et al., 2015).

The use of biochar combined with organic fertilizers can contribute to the increase in microbial taxa related to the cycles of C, N, P, and S, and improve the soil’s ecosystem functions (Antonangelo et al., 2021; Hu et al., 2024). Other combined treatments of biochar at low dosages (0.5 and 2 t ha^-1^) with NPK, cow urine, and organic compost revealed a significant increase of 20%, 103%, and 123%, respectively, in the yield of 13 evaluated crops compared to treatments without biochar (Schmidt et al., 2017). These results are suggested to be due to the high retention capacity and interaction of biochar molecules with urine compounds and fertilizers through chemical bonds and physical entrapment. Puga et al. (2020) demonstrated that the use of 51% biochar with 10% nitrogen (BN_51/10_) increased the yield of maize plants by 21% compared to the control group treated with urea. Furthermore, the BN_51/10_ treatment promoted carbon sequestration in the soil and aided in greenhouse gas mitigation. Another study utilized biochar-urea composites (Bio-MUC) that showed improved N use efficiency and an increase of 37.7% in root volume of maize plants. Additionally, the fresh shoot and root mass increased by 13.8% and 25.1%, respectively, with the Bio-MUC composite (Shi et al., 2020).

### 3.4 Overview of proteomic analysis

**Figure 6:**
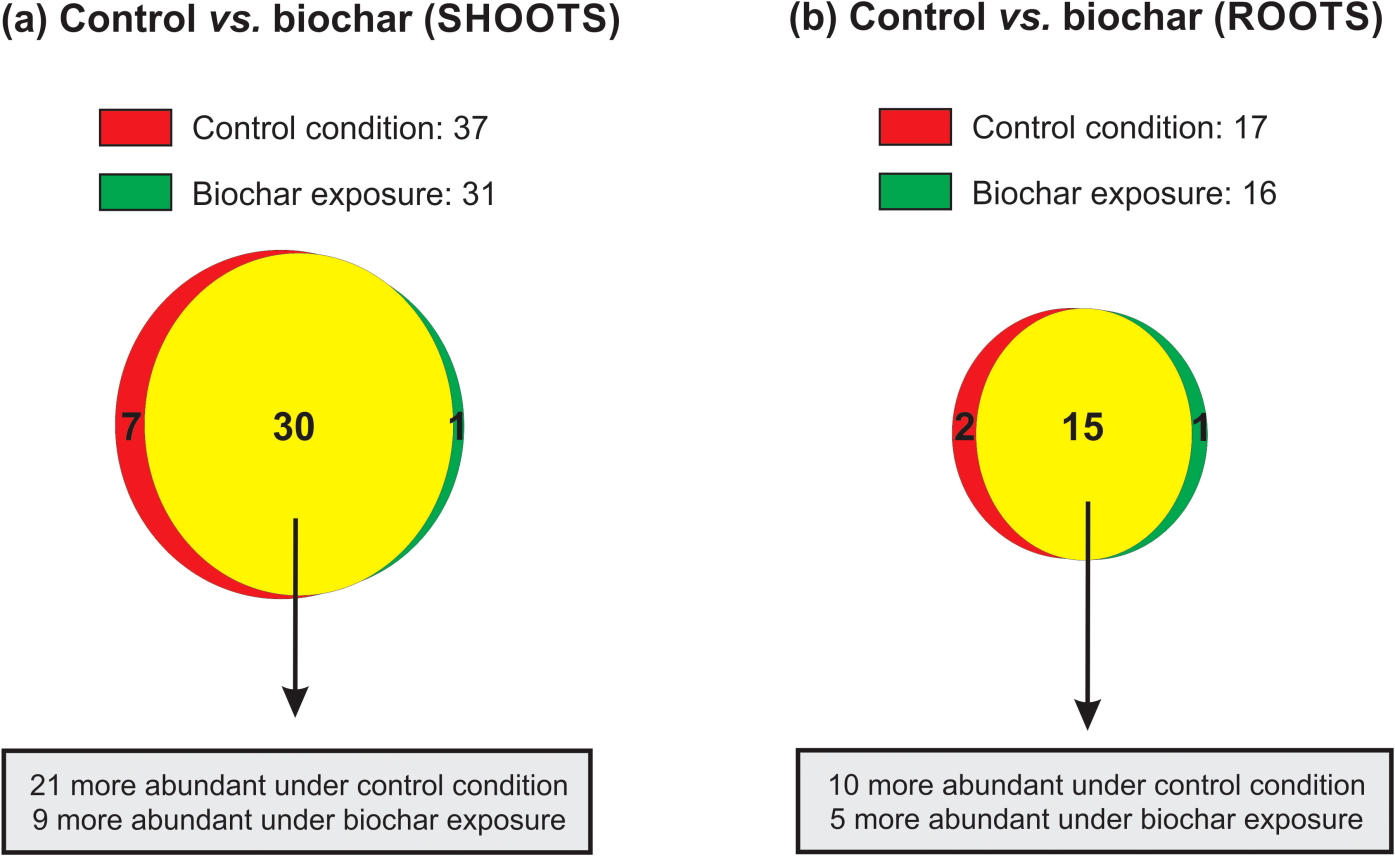
Venn diagram showing the overlap of proteins identified in shoots **(a)** and roots **(B)** of rice plants submitted to control and 0.5 mg mL^-1^ of Spirulina-derived biochar for 20 days. Red circles: control condition; green circle: biochar 0.5 mg mL^-1^; yellow means overlap.

A total of 616 proteins were identified in rice shoots, while 534 proteins were detected in roots, comparing control and biochar (0.5 mg mL^-1^) conditions for 20 days. From these, only 38 (6.2%) and 18 (3.4%) were found exclusively or showed differential abundance in shoots and roots, respectively (**Figure 6**). In the shoots, 21 were more abundant (with 7 unique) under control condition, while 9 were more abundant (with 1 unique) under biochar exposure (**Figure 6A**). In the roots, 10 were more abundant (with 2 unique) under control condition, while 5 were more abundant (with 1 unique) under biochar exposure (**Figure 6B**). Each protein’s sequence underwent comparison with NCBI BLASTp to determine specific domains, molecular functions, and annotations. Afterwards, proteins were classified into functional categories according to existing literature and their presumed molecular functions. **Tables 1** and **2** contain lists of all unique or differentially abundant proteins identified in the rice shoots and roots, respectively. In the following sections, some of these proteins will be discussed in terms of their putative impact on the rice growth/development and the increased seed production observed in biochar-treated plants.

**Table 1:** Differentially abundant proteins in rice shoots after 20 days of exposure to 0.5 mg mL-1 of Spirulina-derived biochar.

Rice Biochar x Control - Proteins unique or more abundant in shoots under control condition					
Functional categories	Description	Uniprot	Reported peptides	t-Test	Log2 Fold Change
**Photosynthesis**	Photosystem II 22 kDa protein 2, chloroplastic	Q0J8R9	3	–	–
	Cytochrome b6-f complex subunit 4	P0C319	6	0.01235	-0.94
	Photosystem I P700 chlorophyll a apoprotein A2	P0C358	12	0.03644	-0.85
	Photosystem II protein D1	P0C434	9	0.00463	-0.65
	Photosystem I P700 chlorophyll a apoprotein A1	P0C355	9	0.03046	-0.63
**Amino acid synthesis**	Threonine synthase	Q6L4H5	4	–	–
	Cysteine synthase	Q9XEA8	7	0.03604	-0.87
**Translation**	Eukaryotic initiation factor 4A-3	Q6Z2Z4	15	–	–
	Elongation factor protein	Q8W0C4	8	–	–
**Cell wall synthesis**	UDP-glucose 6-dehydrogenase 5	Q2QS13	11	–	–
**Protein degradation**	26S proteasome regulatory subunit protein	A0A0P0VG21	2	–	–
**Defense**	Phenylalanine ammonia-lyase	A0A0P0VM80	8	–	–
**Carbohydrate metabolism (Glycogenesis)**	UDP–glucose pyrophosphorylase	Q6ZGL5	8	0.01292	-1.53
**Sulfur transfer**	Thiosulfate sulfurtransferase	Q0D5S1	2	0.01189	-1.35
**Vitamin biosynthesis**	2-methyl-6-phytyl-1,4-hydroquinone methyltransferase 2, chloroplastic	Q2QM69	5	0.02984	-0.71
**Cell cycle**	Rhodanese-like protein	Q6H444	6	0.02216	-0.68

Rice Biochar x Control - Proteins unique or more abundant in shoots under biochar treatment					
Functional categories	Description	Uniprot	Reported peptides	t-Test	Log2 Fold Change
**Translation**	Nascent polypeptide-associated complex alpha subunit	Q8LMR3	3	0.02334	1.18
	Ribosomal protein S6	Q75LD8	2	0.01168	0.94
	Large ribosomal subunit protein uL5c	Q9ZST0	10	0.01971	0.91
	Ribosomal protein L3	Q0E446	5	0.01405	0.84
	30S ribosomal protein S31	Q60EZ7	2	0.01099	0.77
	Ribosomal protein	Q60E59	7	0.01629	0.75
	Rribosomal protein S18	A0A0P0V3F2	2	0.01196	0.73
	Polyprotein of EF-Ts, chloroplastic	Q2QP54	18	0.02363	0.73
	50S ribosomal protein L19	Q6H8H3	2	0.01254	0.62
**Protein modification**	Peptidylprolyl isomerase	Q6ZIT9	4	0.02665	1.51
	Peptidylprolyl isomerase	Q6YW78	9	0.03841	1.29
	Peptidylprolyl isomerase	Q5Z4M6	9	0.04375	1.16
**Photosynthesis/Carbon assimilation**	Carbonic anhydrase	A0A0P0V5S4	2	–	–
	Ferredoxin-1, chloroplastic	Q0J8M2	3	0.04140	1.46
**Photosynthesis**	Photosystem I iron-sulfur center	P0C361	5	0.03461	1.22
	Oxygen-evolving complex protein PsbP	Q2QNI4	2	0.01038	0.64
**Amino acid degradation**	Glycine cleavage system H protein, mitochondrial	A3C6G9	4	0.02589	1.44
	Alanine transaminase	A0A0P0X1V6	23	0.00988	0.65
**Transcription**	S1 RNA binding domain containing protein	Q0DSD6	13	0.04516	1.24
**Protein degradation**	26S proteasome regulatory subunit 6A homolog	P46465	2	0.00912	1.21
**Antioxidant system**	Peroxidase	Q6ER49	9	0.03997	0.94
**Cytoskeleton**	Actin	Q67G20	21	0.02514	0.68

**Table 2:** Differentially abundant proteins in rice roots after 20 days of exposure to 0.5 mg mL-1 of Spirulina-derived biochar.

Rice Biochar x Control - Proteins unique or more abundant in roots under control condition					
Functional categories	Description	Uniprot	Reported peptides	t-Test	Log2 Fold Change
**Stress**	Heat shock 70 kDa protein BIP4	Q75HQ0	2	–	–
	Cold shock domain protein 2	Q84UR8	2	0.00892	-0.92
	Cold shock domain protein 1	Q6YUR8	2	0.04823	-0.66
**Carbohydrate metabolism (Glycolysis)**	Glyceraldehyde-3-phosphate dehydrogenase	Q9SNK3	4	0.03032	-0.89
	Glyceraldehyde-3-phosphate dehydrogenase	Q7X8A1	5	0.04078	-0.84
**Transport**	E1-E2 ATPase domain containing protein	A0A0P0WKS4	4	–	–
**Lipid metabolism**	Non-specific lipid-transfer protein 3	Q2QYL3	2	0.04716	-0.76

Rice Biochar x Control - Proteins unique or more abundant in roots under biochar treatment					
Functional categories	Description	Uniprot	Reported peptides	t-Test	Log2 Fold Change
**Defense**	Hypersensitive-induced reaction protein-like 2	Q6K550	5	–	–
	Jacalin-like lectin domain containing protein	C7J408	4	0.02361	1.87
**Water transport**	Probable aquaporin PIP2-6	Q7XLR1	2	0.02408	1.01
	Aquaporin PIP 1-3	Q9SXF8	3	0.03513	0.78
**Transport**	Plasma membrane ATPase	A0A0P0VQP6	8	0.00109	2.04
**Carbohydrate metabolism (Glycolysis)**	Phosphopyruvate hydratase	A0A0P0WS07	9	0.01182	1.26
**Nucleotide metabolism**	ADP-ribosylation factor protein	Q10QD5	3	0.02475	0.68
**Phenylpropanoid biosynthesis**	Trans-cinnamate 4-monooxygenase	Q5W6F1	2	0.03379	0.67
**Translation**	Elongation factor protein	A0A0N7KIH5	13	0.02630	0.60
**Others**	Transferase family protein protein	A0A0P0Y2T0	5	0.00842	0.99
	Dehydrogenase protein	A0A0P0VRW1	4	0.04013	0.62

#### 3.4.1 Shoot proteins affected by biochar

Surprisingly, after 20 days of exposure to biochar, the functional category most inhibited in rice shoots was photosynthesis, as evidenced by decreased abundance of proteins such as Photosystem II 22 kDa protein 2, Cytochrome b6-f complex subunit 4, Photosystem I P700 chlorophyll a apoproteins, and Photosystem II protein D1. Furthermore, proteins involved in amino acid synthesis (Threonine and Cysteine synthase), cell wall synthesis (UDP-glucose 6-dehydrogenase 5), defense (Phenylalanine ammonia-lyase), glycogen synthesis (UDP-glucose pyrophosphorylase), sulfur transfer (Thiosulfate sulfurtransferase), vitamin biosynthesis (2-methyl-6-phytyl-1,4-hydroquinone methyltransferase 2), and cell cycle (Rhodanese-like protein) were also detected as less abundant in the shoots of rice plants under biochar treatment (**Table 1**). This down-regulation of processes under biochar treatment suggests that rice plants may adjust growth, development, and biosynthetic processes during the vegetative stage to optimize resource allocation towards other energy-demanding processes, such as seed production. The reduced energy demand resulting from decreased biosynthetic processes may prompt the replacement of PSI and PSII proteins, thereby adjusting the plant’s photosynthetic capacity to match its energy requirements (Li et al., 2018). Additionally, by reducing the investment in biosynthesis, plants may prioritize resources towards root biomass accumulation, as evidenced by the high root dry weight (**Figure 4D**), despite the observed decrease in root length (**Figure 4B**) after 20 days of exposure to biochar. The decrease in root length observed in response to biochar treatment could potentially be associated with the reduced abundance of the Rhodanese-like protein, a superfamily that fulfills various cellular functions ranging from resistance to environmental threats such as cyanide, to key cellular reactions related to sulfur metabolism and progression of the cell cycle (Cipollone et al., 2007), both processes also inhibited by the biochar treatment in our study.

However, it’s important to note that such a strategy of reducing key processes during the vegetative stage in order to optimize resource allocation towards the reproductive stage would likely be feasible only under non-stressed environments (and with well-nourished plants), since most of the energy produced under challenging growing conditions is typically allocated to defense/stress-responsive pathways (Caretto et al., 2015). Initially, the down-regulation of primary metabolic processes under biochar treatment could be interpreted as a negative effect, as reported in different studies (Liao et al., 2014; Gale et al., 2016; Liu et al., 2021a). However, most of these studies focus solely on physiological responses immediately after biochar treatment, without considering the entire plant development or analyzing seed production.

On the other hand, the functional category most induced in rice shoots by biochar exposure was translation (Nascent polypeptide-associated complex alpha subunit, Ribosomal proteins, and Polyprotein of EF-Ts). Proteins related to protein modification (Peptidylprolyl isomerase), carbon assimilation (Carbonic anhydrase), amino acid degradation (Glycine cleavage system H protein and Alanine transaminase), transcription (S1 RNA binding domain containing protein), antioxidant system (Peroxidase), and Cytoskeleton (Actin) were also detected as more abundant in the shoots of rice plants under biochar treatment (**Table 1**). Notably, biochar treatment enhances translational capacity and the flux of nascent proteins in rice shoots. It is already known that under non-stress conditions, the Nascent polypeptide-Associated Complex (NAC) associates with ribosomes to promote translation and protein folding (Kirstein-Miles et al., 2013). Peptidyl-Prolyl Isomerases (PPIs) play roles in the folding of newly synthesised proteins (Shaw, 2002), and several proteins with PPI activity have been implicated with plant stress defense/tolerance (Lee et al., 2015; Yoon et al., 2016; Roy et al., 2022). Rice plants overexpressing *OsCYP19-4*, a cyclophylin with peptidyl-prolyl cis-trans isomerase activity, show an increase in grain yield (Yoon et al., 2016).

While there may be studies exploring the role of carbonic anhydrase (CA) activity in various aspects of plant physiology, including photosynthesis and carbon fixation (Rudenko et al., 2021), there is no study directly linking CA activity to improved seed production in plants. However, it’s important to note that the role of CA in facilitating CO_2_ assimilation and pH regulation within plant cells (DiMario et al., 2018) could indirectly influence seed production by optimizing photosynthetic efficiency and overall plant growth. Additionally, increased CA activity may promote C assimilation and partitioning towards reproductive sinks, including developing grains, by providing a steady supply of C precursors that can support grain filling and increase grain yield in crop plants (Julius et al., 2017). Glycine and Alanine degradation pathways are interconnected with central C and energy metabolism. The breakdown of these amino acids can generate intermediates that enter energy-producing pathways, such as the tricarboxylic acid cycle, to produce ATP (Hildebrandt et al., 2015). Increased energy availability resulting from Glycine and Alanine degradation may therefore support the energy-demanding processes associated with protein translation, such as ribosome biogenesis and aminoacyl-tRNA charging.

Besides being key components of the plant antioxidant defense system, which helps maintain cellular redox homeostasis by detoxifying ROS (Freitas et al., 2024), peroxidases are involved in cell wall remodeling processes, including lignification and cross-linking of cell wall components (Barros et al., 2015). Enhanced peroxidase activity can promote cell wall strengthening and rigidity, which can provide structural support to developing plant tissues, including the vascular system. This enhanced structural integrity can reduce lodging and support efficient nutrient and water transport (Notaguchi and Okamoto, 2015), contributing to increased grain filling and yield.

#### 3.4.2 Root proteins affected by biochar

The functional category most inhibited in rice roots after 20 days of exposure to biochar was stress (Heat shock 70 kDa protein BIP4 and Cold shock domain proteins). One protein related to lipid metabolism (Non-specific lipid-transfer protein 3) was also detected as less abundant in the roots of rice plants under biochar treatment (**Table 2**). Plant non-specific lipid-transfer proteins (nsLTPs) are involved in key processes, including resistance to biotic/abiotic stress and plant growth/development (Missaoui et al., 2022). Once again, the reduced accumulation of stress-related proteins under biochar treatment appears to be part of an energy reallocation strategy, wherein certain energy-demanding processes are downregulated in favor of others. Since the control plants were not under stressful conditions, we believe that a higher abundance of stress-related proteins in this condition than biochar-treated ones does not necessarily represent a stressful condition, but rather a constitutive response that can be finely controlled (decreased) in cases where the plant needs to reallocate energy resources.

On the other hand, the functional categories most induced in rice roots by biochar exposure were defense (Hypersensitive-induced reaction protein-like 2 and Jacalin-like lectin domain containing protein) and water transport (Probable aquaporin PIP2-6 and Aquaporin PIP1-3). Proteins related to phenylpropanoid biosynthesis (Trans-cinnamate 4-monooxygenase), and translation (Elongation factor protein) were also detected as more abundant in the roots of rice plants under biochar treatment (**Table 2**). According to Li et al. (2019), the *hypersensitive induced reaction 3* (*HIR3*) gene contributes to rice basal resistance, and Nadimpalli et al. (2000) postulated that these proteins are generally involved in controlling ion channels, particularly potassium ion channels. Considering that we found potassium minerals in the Spirulina-derived biochar composition, we hypothesize that the hypersensitive-induced reaction protein-like 2 found in our work could be related to potassium transport. Nonetheless, the higher abundance of two defense-related proteins under biochar exposure seems to indicate that these plants shift their basal protection from stress proteins to defense proteins.

It is widely acknowledged that biochar application improves water uptake and holding capacity (Wu et al., 2023), and thus the use of biochar as a soil amendment can be a worthy strategy to guarantee yield stability under short-term water-limited conditions (Carvalho et al., 2014). Aquaporin proteins belong to a major intrinsic protein superfamily that has a crucial role in transporting water and some other molecules (Soliman et al., 2023). Therefore, the higher abundance of these proteins under biochar treatment was not unexpected. The phenylpropanoid pathway is universally acknowledged as a crucial supplier of metabolites in plants, pivotal for lignin synthesis and as a precursor for numerous significant compounds such as flavonoids, coumarins, and lignans (Fraser and Chapple, 2011). Furthermore, phenylpropanoids play essential roles in various facets of plant physiology, including growth, development, and responses to environmental challenges, while also functioning as potent antioxidants (Rahim et al., 2023). Accordingly, we propose that rice plants treated with Spirulina-derived biochar exhibit greater diversity in secondary metabolites than control plants.

### 3.5 Computational systems biology analyses

**Figure 7:**
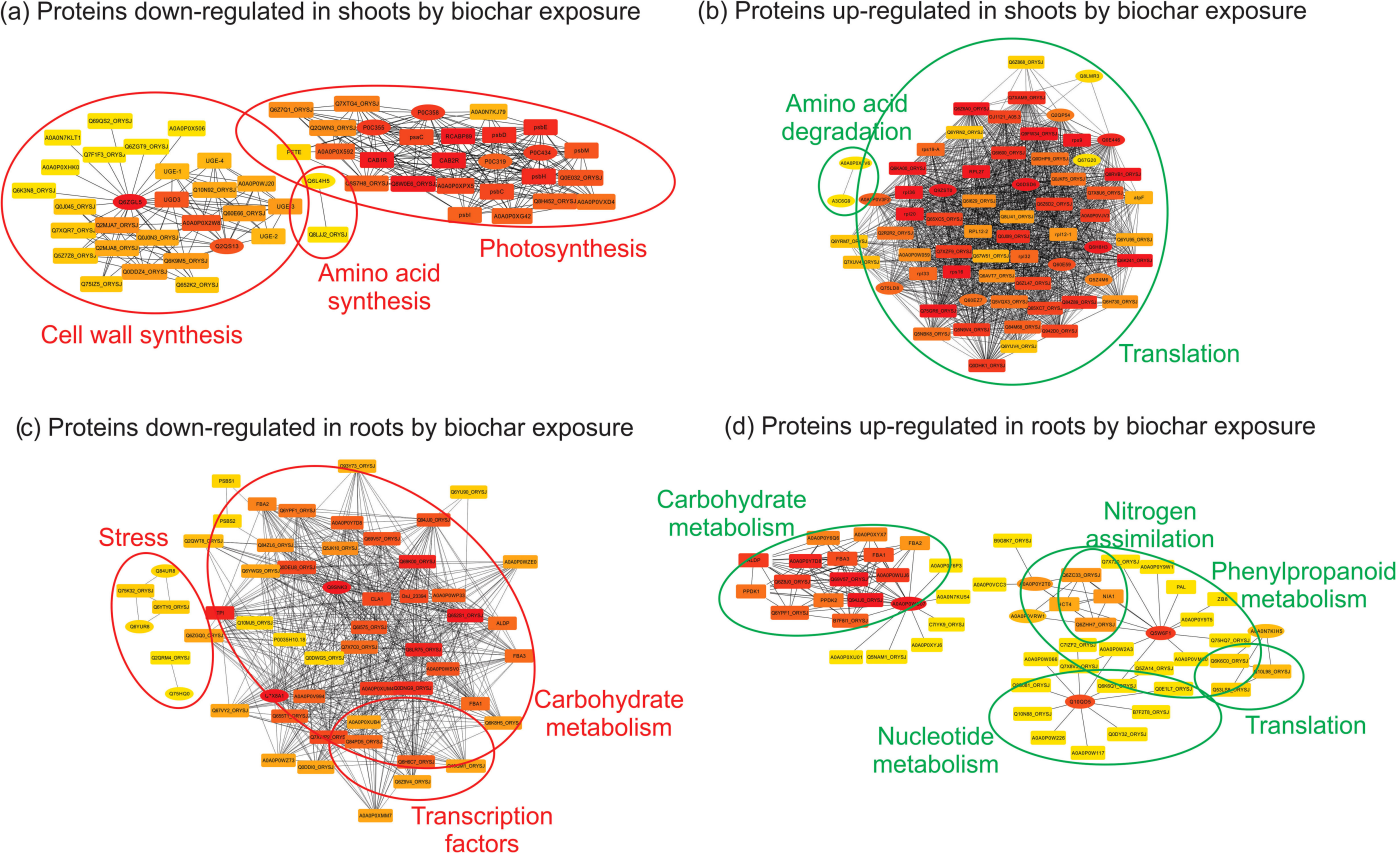
Graphical visualization of the protein-protein interaction (PPI) network generated including proteins with lower **(A, C)** and higher **(B, D)** abundance in shoots **(A, B)** and roots **(C, D)** of rice plants exposed to 0.5 mg mL^-1^ of Spirulina-derived biochar for 20 days, when compared to control condition. Rectangles nodes represent the shell proteins directly associated with our input proteins (ellipsis nodes), while edges (straight lines) represent known or inferred interactions. The network structure derives from Cytoscape following the application of the Organic layout. The color indicates the degree value (red indicates greater degree).

Protein-protein interaction (PPI) networks containing the proteins with lower (down-regulated) and higher (up-regulated) abundance in rice shoots and roots after 20 days of exposure to 0.5 mg mL^-1^ of biochar are shown in **Figure 7**. Corroborating the data presented in **Table 1**, exposure of rice plants to biochar inhibited biosynthetic processes in shoots, such as photosynthesis, cell wall synthesis, and amino acid synthesis (**Figure 7A**), and stimulated shoot translation and amino acid degradation (**Figure 7B**). In the roots, besides the previously detected functional categories shown in **Table 2**, the PPI interaction analysis revealed two novel metabolic processes modified by biochar exposure: inhibition of transcription factors (**Figure 7C**), and stimulation of nitrogen assimilation (**Figure 7D**). Both modified processes in rice roots during the vegetative stage may be relevant for increasing grain production. With decreased activity of transcription factors in root cells, less energy is expended on regulating gene expression related to root development, allowing more resources to be allocated to other processes such as grain formation and growth (Vain et al., 2023). Also, transcription factors are involved in regulating carbohydrate metabolism, and a decrease in their activity can positively affect starch reserve accumulation in grains, which is essential for their growth and development (MacNeill et al., 2017). It is interesting to note that a small number of proteins related to carbohydrate metabolism were activated in rice roots after biochar exposure (**Figure 7D**), but the amount of carbohydrate metabolism-related proteins inhibited by biochar exposure was much greater (**Figure 7C**), evidencing a downward remodeling of this pathway. At the same time, increased nitrogen assimilation in root cells is important to sustain the increased translation detected in both tissue (shoots and roots) under biochar exposure (**Figures 7B, D**). Nitrogen assimilation in the roots can enhance the plant’s ability to absorb other essential nutrients from the soil, such as phosphorus and potassium (Anas et al., 2020; Jiaying et al., 2022). This improved nutrient uptake can contribute to overall plant vigor, facilitating better grain development and yield (Zhang et al., 2022).

## 4 Conclusion

**Figure 8:**
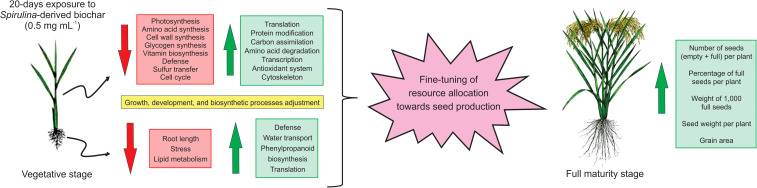
Schematic model illustrating the rice responses after 20 days of exposure to biochar derived from *Arthrospira platensis*, and its subsequent impact on seed production.

The biochar obtained from the pyrolysis of *Arthrospira platensis* biomass has emerged as a promising solution for enhancing rice seed production. With an impressive yield of 55% and key attributes such as the presence of mineral salts and a porous structure, it has the potential to facilitate nutrient and water adsorption and gradual release. Exposure of rice plants to 0.5 mg mL^-1^ of biochar for 20 days resulted in alterations in the expression of numerous proteins in both shoots and roots, indicating, in conjunction with the physiological findings, a fine-tuning of resource allocation towards seed production. These alterations are summarized in **Figure 8**. Consequently, this product holds promise as a viable biostimulant for augmenting rice productivity. It would be interesting to test whether Spirulina-derived biochar can also be used to increase seed production in other plant species cultivated under different agricultural practices and water regimes, thus offering a significant contribution to sustainable agriculture and global food security.

## Data Availability

The datasets presented in this study can be found in online repositories. The names of the repository/repositories and accession number(s) can be found in the article/supplementary material.
